# Factors influencing the decision of GHANAIAN optometry students to practice in rural areas after graduation

**DOI:** 10.1186/s12909-018-1302-3

**Published:** 2018-08-06

**Authors:** Samuel Bert Boadi-Kusi, Samuel Kyei, Vandyke Bright Okyere, Sampson Listowell Abu

**Affiliations:** 1 Department of Optometry and Vision Science, School of Allied Health Sciences, College of Health and Allied Sciences, University of Cape Coast, Cape Coast, Ghana; 2 Department of Optometry, Faculty of Health Sciences, Mzuzu University, Mzuzu, Malawi; 3 Department of Ophthalmology and Visual Sciences, School of Medicine, University of Alabama at Birmingham, Birmingham, AL USA

**Keywords:** Ghanaian optometry students, Rural area, Incentives, Optometric practice

## Abstract

**Background:**

Human resources for eye health are inequitably distributed in most developing countries including Ghana. In spite of this, most eye care workers are concentrated in urban areas to the disadvantage of rural dwellers who need the services of these workers the most. The aim of the study was to investigate factors that will influence Ghanaian Optometry students’ decision to work in rural areas after completion of their training.

**Method:**

A cross-sectional survey was conducted among Ghanaian optometry undergraduate students. All undergraduate optometry students (first to sixth year) who agreed to take part in the research completed a 37 item questionnaire that explored; demographic characteristics, views about practice choice and possible attractions and incentives to practice in the rural area.

**Results:**

A total of 333 (87.4%) participants out of 381 Ghanaian optometry students who were registered for the 2015/2016 academic year completed the questionnaire. Rural origin students had the greatest desire to practice in the rural setting when employed by the Government (78.9%) or by NGO (80.3%). Financial incentives (76.6%), scholarship for further studies (76.0%), better living conditions (71.2%) and career ladder jump for rural health workers (71.2%) were the main incentives that influenced the intention of graduate optometrists to practice in the rural areas.

**Conclusion:**

Rural origin students are more inclined to work in rural areas than urban origin students, a finding which is informative for optometry training schools when managing their admission policies. Financial incentives among other factors will encourage more students to engage in rural optometric practice irrespective of their place of origin.

## Background

Globally, 285 million people are estimated to be visually impaired, of whom 39 million are blind and 246 million living with low vision [1]. Although about 80% of the causes of visual impairment are preventable, researchers have projected a rise in the prevalence of visual disorders particularly for Sub-Saharan Africa due to poor access to eye care services [1, 2]. With just 11% of the global population, Sub-Saharan Africa is plagued with about 24% of the global burden of visual impairment and blindness. The nations within this region have the least number of eye care professionals and do not meet the minimum human resources requirement of one eye health professional for every 55,000 people [3]. This imbalance together with limited eye care facilities contribute immensely to the high prevalence of visual impairments observed in the region [3, 4].

The burden of limited access to eye care is further compounded by the uneven distribution of eye care professionals between rural and urban settlements. About 90% of people with visual impairment live in low income and rural settings [1] where there are an insufficient number of healthcare facilities and human resources for eye health [5]. Eye care services in Sub-Saharan Africa, including Ghana, are provided mainly by optometrists and ophthalmologists who are usually drained to urban areas. This results in insufficient number of qualified ophthalmic personnel attending to the overwhelming eye care needs in the rural areas [6, 7]. In a previous study, we found that the majority (71.1%) of optometrists in Ghana practiced in urban areas [7]. The cause of uneven distribution of health care professionals is multifaceted: better incomes, more opportunities for career progression, better infrastructure and improved access to social amenities [8, 9].

The World Health Organization (WHO), the International Agency for the Prevention of Blindness (IAPB), non-governmental organizations (NGOs) and other eye care stakeholders enacted the global Vision 2020 initiative with the ultimate goal of eliminating preventable blindness by the year 2020 [10]. Strategies to achieve this goal have included improving access to eye care in rural areas and the training of more eye care practitioners (optometrists and ophthalmologists) with emphasis on provision of refractive services, vision rehabilitation and cataract surgeries. Optometrists have competencies to offer services such as refractive error correction by the provision of spectacle lenses and contact lenses, pediatric vision management, binocular vision anomaly management, low vision rehabilitation, and diagnoses of ocular diseases and management [11]. The role played by optometrists in eye care delivery is core to realizing Vision 2020.

On the other hand, training of more optometrists would not necessarily translate into improved access to eye care services, if there are no existing policies to attract and retain them in the rural communities [12, 13]. Like other health care professionals, attracting eye care professionals to rural areas is a challenge facing many countries, both developed and developing. In a South African study, Mashige and his colleagues found that majority of optometry students would not consider starting their first practice in a rural area [9].

While it may be difficult for optometrists in urban areas to relocate to rural communities, implementing strategic initiatives targeting new optometry graduates could address the disparity in the distribution of optometrists in Ghana. Such initiatives could be well formulated and be effective when based on known factors which influence trainees’ decision on rural practice. As a follow up to our previous study [7] and to serve as a potential reference for policy development, we investigated the factors that would influence the decision of Ghanaian optometry students about working in a rural setting after completion of their training.

## Method

### Study design

**Fig. 1:**
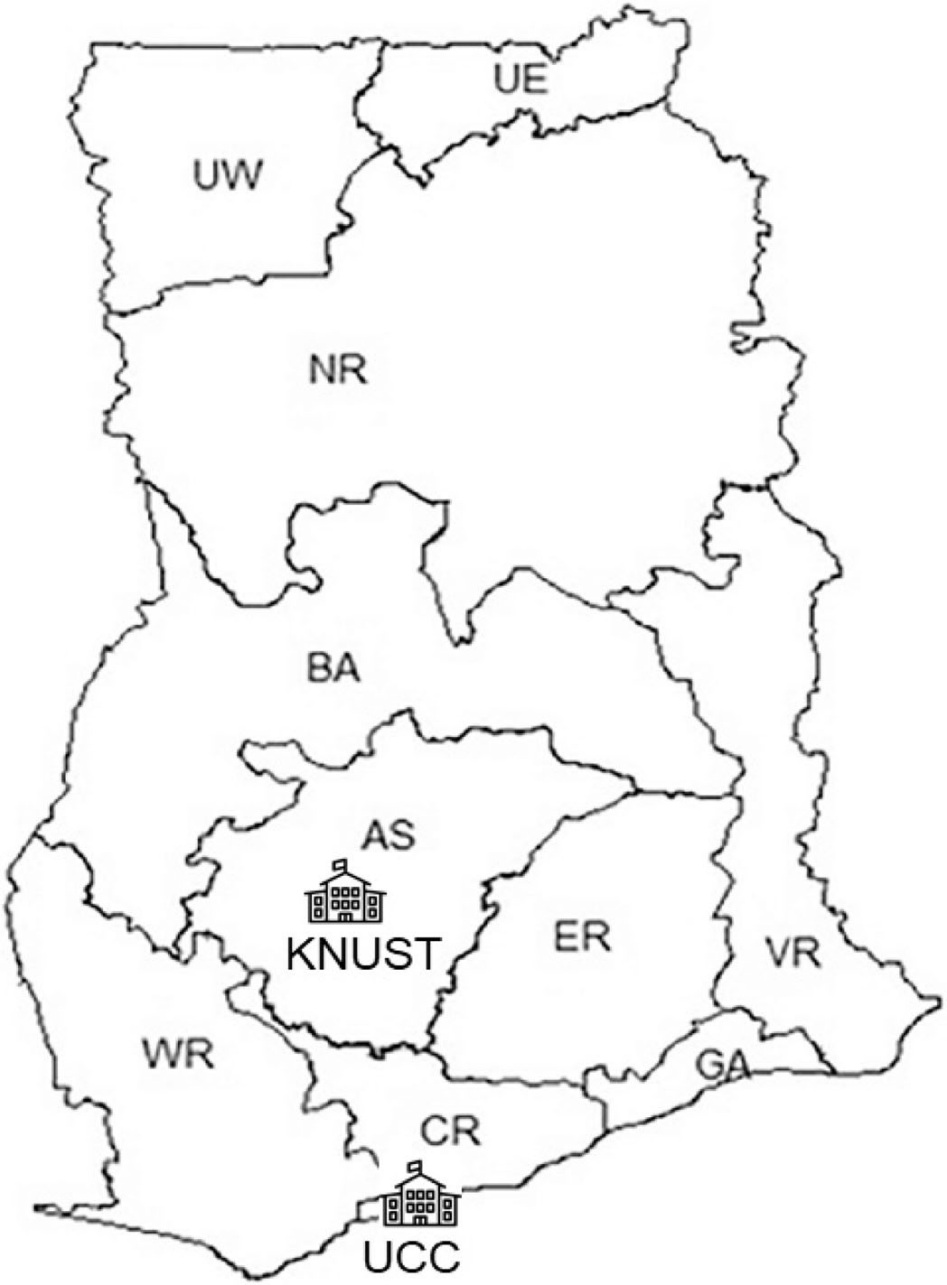
The map of Ghana showing the location of the two optometry training institutions and the ten administrative regions. UE: Upper East UW: Upper West NR: Northern Region BA: Brong Ahafo AS: Ashanti ER: Eastern Region VR: Volta Region GA: Greater Accra CR: Central Region WR: Western Region

This study was a quantitative cross-sectional survey of optometry students in the two training institutions in Ghana; University of Cape Coast (UCC) and Kwame Nkrumah University of Science and Technology (KNUST), (Fig. 1). The study included the first year to sixth-year students enrolled in the 2015/2016 academic year. The list of enrolled students was obtained from the respective schools and only students with Ghanaian nationality were invited to participate in the study. Ethical approval for the study was obtained from the Institutional Review Board of the University of Cape Coast. We did not collect personal identifying information such as name or hometown to ensure anonymity. The study adhered to the other tenets of the Helsinki Declaration and only consenting students participated in the study.

### Data collection

This study adopted a validated questionnaire previously used in a related study in South Africa [9]. The questionnaire was modified to suit the Ghanaian setting. It comprised 37 open and closed-ended questions that explored demographic characteristics, views about practice choice, possible attractions, and incentives among others. Eligible students took approximately 30 min to complete the self-administered paper-based questionnaires in English. For the purpose of this study, a rural area was defined as an area with a population size cut off of less than 5000 with its inhabitants mainly engaged in primary (agricultural) activities [14]. Although this definition has a lot of shortcomings as admitted by the Ghana Statistical Services, the authors adopted it because that is the definition being used currently in all Ghanaian official documentation [14].

### Data analysis

Data were captured and analyzed with the Statistical Package for the Social Sciences (SPSS), version 21.0 (Chicago, IL, USA). Descriptive statistics such as frequencies and percentages and cross-tabulations were used to analyze the data. Binary logistic regression was used to evaluate the likelihood or willingness of students of different backgrounds to practice in rural areas. The Friedman test was used to assess whether various motivating factors influenced the choice of rural practice differently. Differences in test were considered significant if *p* < 0.05.

## Results

### Demographic profile

A total of 333 (87.4%) out of the 381 enrolled Ghanaian optometry students in both institutions {193(58%) at UCC and 188(42%) at KNUST)} completed the questionnaire. The mean age of participants was 22.67 ± 2.03 and males constituted majority of the participants (*n* = 245, 73.6%). Although more than half of the respondents (*n* = 181, 54.4%) come from urban communities, this proportion was not significantly greater than (*p* = 0.112) those of rural background (*n* = 152, 45.6%). There was representation from all the 10 administrative regions of the country, those with the smallest numbers being from the three northern regions. Detailed socio-demographic characteristics of study participants are shown Table 1.

**Table 1:** Demographics of 2015/2016 Ghanaian Optometry Students

Characteristics	*N* = 333	%
Age		
17–19	23	6.9
20–22	118	35.4
> 22	192	57.7
Gender		
Male	245	73.6
Female	88	26.4
Marital Status		
Single	328	98.5
Married	5	1.5
Place of origin		
Urban	181	54.4
Rural	152	45.6
Institution of Study		
UCC	173	52.0
KNUST	160	48.0
Region of Origin		
Greater Accra	26	7.8
Ashanti	94	28.2
Eastern	51	15.3
Volta	33	9.9
Western	24	7.2
Central	53	15.9
Brong Ahafo	30	9.0
Northern	12	3.6
Upper East	6	1.8
Upper West	4	1.2
Year of Study		
1st	81	24.3
2nd	41	12.3
3rd	67	20.1
4th	66	19.8
5th	26	7.8
6th	52	15.6

### Description of participants with rural background

**Fig. 2:**
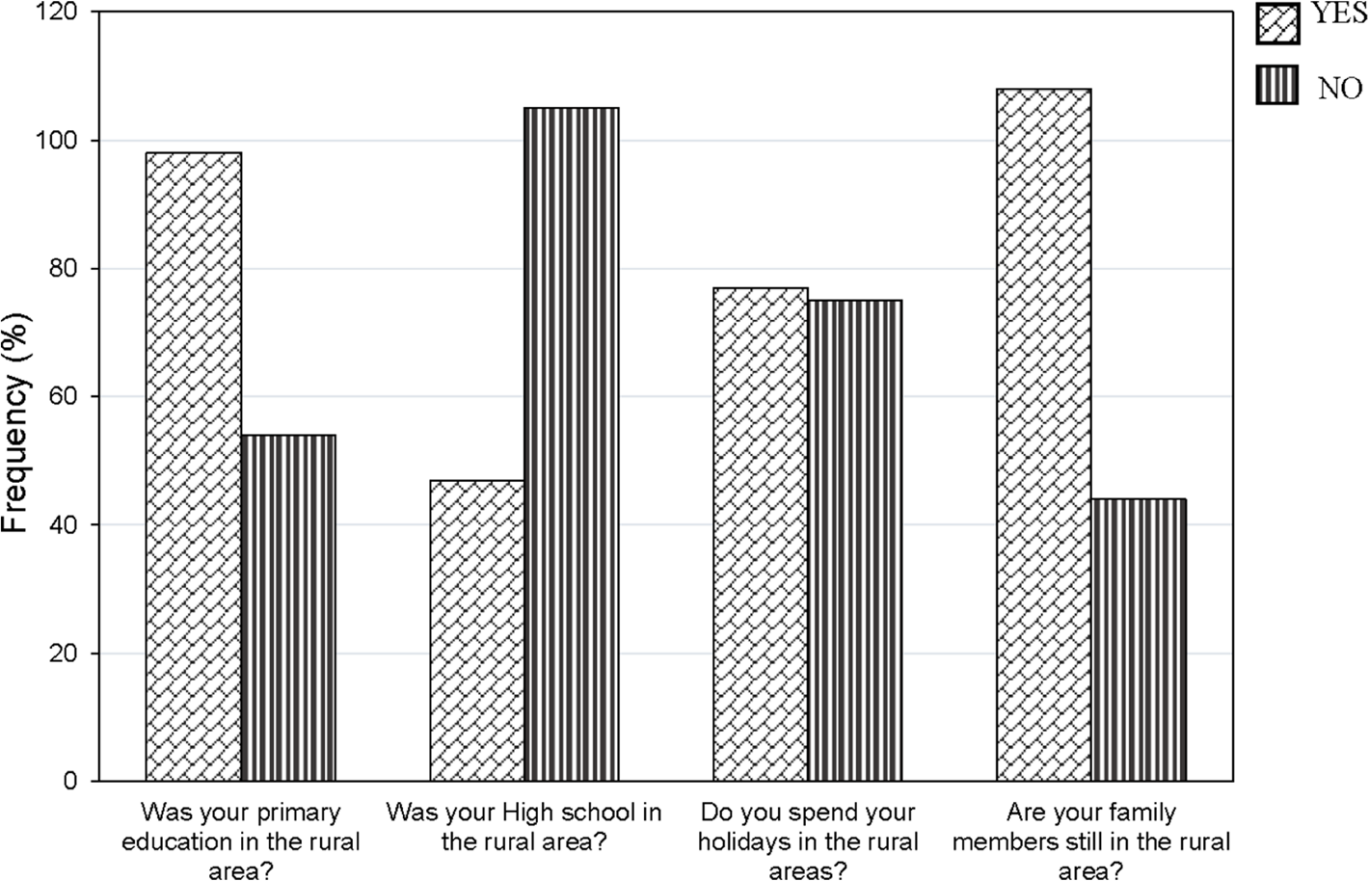
Characteristics of respondents from rural areas

Of the participants from rural background, 98(64.5%) had their primary/basic education in rural areas, however, over two-thirds (*n* = 105, 69.1%) attended high school in urban areas. Figure 2 presents additional details regarding the percentages of those who returned to their rural communities for holidays and those having relatives who live in rural communities.

### Views about practicing in rural areas

The majority of the respondents would accept postings to the rural areas from either the Government (*n* = 243; 75.2%) or an NGO (*n* = 258; 79.9%) (Table 2). Approximately half of the respondents (*n* = 169; 52.0%), of which 103 were students of urban backgrounds, (*n* = 103; 56.9%) opposed mandatory postings to rural communities. A significant number of the students (*n* = 217; 65.8%) were unwelcoming of the idea to establish their first practice in a rural area. Nonetheless, most of these respondents would consider establishing their subsequent (*n* = 188; 86.6%) practice in a rural area. Comparing responses, students of urban background were more likely to reject opportunities to work in rural areas (Table 3).

**Table 2:** Respondents view about working in Rural areas

Variables	Response	Rural N (%)	Urban N (%)	Total N (%)
Establish first practice in rural area.	YES	64(42.1)	52(28.7)	116(34.8)
	NO	88(57.9)	129(71.3)	217(65.2)
Establish subsequent practice in the rural area	YES	81(92.0)	107(82.9)	188(86.6)
	NO	7(8.0)	22(17.1)	29(13.4)
^a^Employed by the Government	YES	120(80.6)	123(70.7)	243(75.2)
	NO	29(19.4)	51(29.3)	80(24.8)
^a^Employed by an N.G.O	YES	122(83.0)	136(77.3)	258(79.9)
	NO	25(17.0)	40(22.7)	65(20.1)
^b^Compulsory posting of graduates to rural areas	YES	81(55.1)	75(42.1)	156(48.0)
	NO	66(44.9)	103(57.9)	169(52.0)

^a^ 10 non-respondents ^b^ 8 non-respondents

**Table 3:** Odds for students of urban background to reject working in rural area in reference to those of rural backgrounds

Variables	Odds ratio	95% CI		*p*- value
		Lower boundary	Upper boundary	
Establish your first practice in a rural area	1.80	1.14	2.85	0.01
Employed by the Government to work in a rural area	1.72	1.02	2.89	0.04
Employed by an NGO to work in a rural area	1.44	0.82	2.50	0.20
Compulsory posting to rural areas by the Government	1.68	1.09	2.62	0.02

Rural background used as the reference

### Motivating factors and the choice of rural practice

**Fig. 3:**
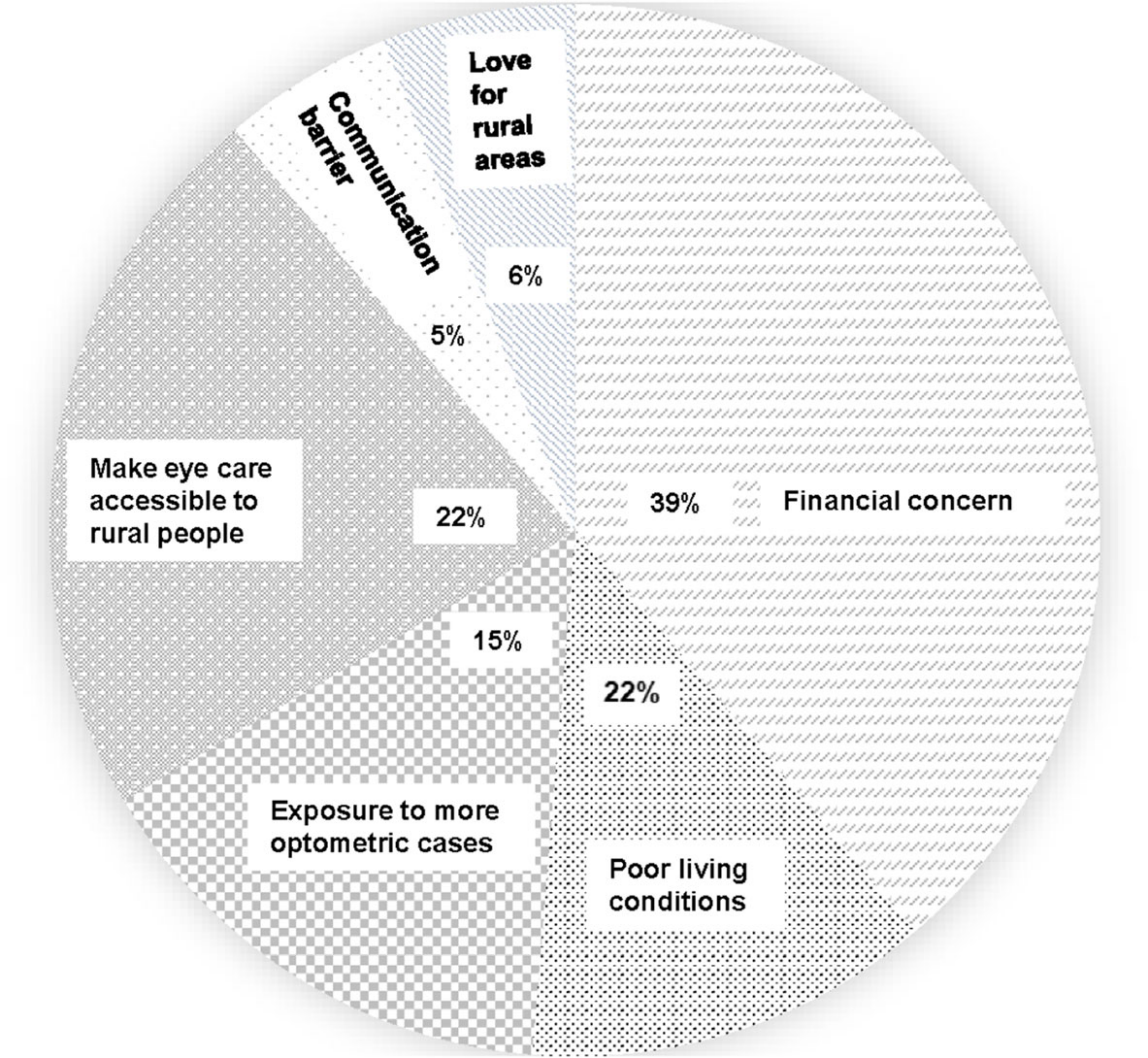
Factors that would be considered by respondents when deciding on rural practice

Financial concern (*n* = 130; 39.0%) was the most common among other factors that would be considered by respondents when making decisions about practicing in rural areas (Fig. 3). The ranking of various motivating factors associated with choosing rural practice has been provided in Table 4. Good Salary was the highest ranked factor with the lowest Friedman mean rank of 2.10, followed by ‘Additional Incentives’ with a mean rank of 2.32. ‘Love for rural areas’ was the least ranked motivation factor. There were significant differences in the associations between motivating factors and choosing rural practice (χ2 = 231.224, *p* = 0.001).

**Table 4:** Motivating factors and choosing to work in the rural area

Variable	Friedman Mean Rank	χ^2^	df.	*p*-value
Good salary	2.10	2311.22	4	< 0.001
Additional incentives	2.32			
No job in the rural centers	3.49			
Proximity to home	3.50			
Love for rural areas	3.59			

### Incentives that would attract respondents to rural optometric practice

A significant proportion of the respondents indicated that financial incentives (*n* = 237; 71.2%), scholarship for further studies (*n* = 253; 76.0%), better living conditions (*n* = 237; 71.2%) and career ladder jump for rural health workers (*n* = 237; 71.25) were the most enticing incentives that would draw them to rural areas (Table 5).

**Table 5:** Incentives that will attract fresh graduates to engage in rural optometric practice

Incentives	NAA N(%)	NRA N(%)	SA N(%)	MA N(%)	VA N(%)	MA + VA N(%)
Facilities for professional development	41(12.3)	19(5.7)	34(10.2)	78(23.4)	152(45.6)	230(69.0)
Scholarship for further studies	28(8.4)	15(4.5)	31(9.3)	72(21.6)	181(54.4)	253(76.0)
Better living conditions	30(9.0)	22(6.6)	35(10.5)	65(19.5)	172(51.7)	237(71.2)
Financial incentives	34(10.2)	10(3.0)	28(8.4)	65(19.5)	190(57.1)	255(76.6)
Acceptable standard working place	29(8.7)	21(6.3)	46(13.8)	71(21.3)	158(47.4)	229(68.7)
Outreach interaction between rural and urban health workers	32(9.6)	32(9.6)	63(18.9)	64(19.2)	134(40.2)	198(59.4)
Career ladder jump for rural health workers	26(7.8)	31(9.3)	33(9.9)	70(21.0)	167(50.2)	237(71.2)
Facilities of knowledge exchange to reduce sense of professional isolation	32(9.6)	40(12.0)	58(17.4)	71(21.3)	125(37.5)	196(58.8)
Enhanced profile of rural health workers.	31(9.3)	37(11.1)	60(18.0)	79(23.7)	120(36.0)	199(59.7)
Mandatory community service	58(17.4)	37(11.1)	72(21.6)	71(21.3)	89(26.7)	160(48.0)

*NAA* Not Attractive at All, *NRA* Not Really Attractive, *SA* Slightly Attractive, *MA* Moderately Attractive, *VA* Very Attractive

## Discussion

Shortage of healthcare workers in rural and remote areas remains a growing concern both in developed and developing countries. In the face of growing eye care needs and high prevalence of visual impairment, Ghana battles acute shortage of optometrists in rural areas. This study sought to find out what factors optometry students would consider when deciding on the location of their prospective practices. More than half of the study respondents came from urban areas, however students with rural backgrounds were more inclined to return and work in rural areas. The findings also indicated that employment by government and NGOs would attract optometry graduates to rural areas despite their place of origin. The majority of Ghanaian optometry students, irrespective of their background, would, however, not start their first practices in rural communities. In addition, we found that financial concern was the main hindrance for the optometrists to opt for rural practice.

The study found a similar proportion of respondents (65.2%) as reported by Mashige et al. (66%) [9] who would not consider opening their first practice in rural areas. The South African study [9] and this current study are comparable in terms of similar response rate and mean age of respondents, however, the former had more female participants (67%) than this study (26.4%). Boadi-Kusi et al. reported under-representation of females in both the optometric workforce and training institutions in Ghana [7, 15]. The Ghanaian optometry curriculum spans a 6-year duration, two years longer than South African. The smaller number of female optometry students may be attributed to the fact that males tend to be more interested in natural sciences [16], decreased enrollment of females into tertiary institutions [16] as well other cultural pressures such as early marriage [17, 18]. In their study of Ghanaian medical students, Agyei-Baffour et al. also found that respondents were not motivated to work in rural areas [19]. Similar findings among Ghanaian optometry students appear to corroborate the fact that students enrolled in healthcare programs are more likely to end up practicing in urban areas. This would further aggravate the burden of limited access to health care in rural communities.

Government and NGOs are the largest providers of health services in Ghana, particularly in rural communities [20]. The results of this study showed that about two-thirds of students, irrespective of their background, would accept rural employment or posting from the government and NGOs. This observation is consistent with the findings by Mashige and his colleagues [9]. The government and NGOs can, therefore, increase access to eye care in rural communities by pursuing policies of setting up community-based eye units, beyond the district level, and posting optometrists to these communities [21].

A greater percentage of students enrolled in most healthcare training programs such as optometry and medicine tend to come from urban areas [9, 15, 19] and are usually unreceptive of rural employment. It is important to underscore that our finding compliments the 2010 national census of Ghana, which found that a little over half of Ghana’s population (50.9%) lives in urban localities. There is, however, no uniformity across all the regions in the country as only two regions (Greater Accra and Ashanti) contributed to this drift [14]. The current study’s finding that students of rural backgrounds were more likely to practice in rural areas than those of urban background, may stem from the fact that students from rural areas have a better understanding and firsthand experience of the eye care plight facing their people [22]. Optometry training intuitions in Ghana may also adopt a policy of reserving a quota of their admission slots for students of rural backgrounds [9]. The goal of such as a policy will be to train and motivate them to return to their rural communities and improve access to eye care.

The commonly expressed reasons for choosing an optometry profession just like any other healthcare professions include a desire to help others, job availability, and prestige [15, 19, 23]. It is, therefore, important to identify competing factors that prevent healthcare professionals from practicing in rural areas that face more deplorable health conditions [8, 21, 24]. This study found financial concern as the most common factor which optometry students would consider when deciding on working in a rural setting. Financial concern has been reported as a major hindrance to rural practice in studies involving optometry students in South Africa [9], nurses [25] and physicians [26]. Extrinsic motivation has been found to be highly associated with rural practice choice than intrinsic motivation [19].

Toward increasing access to eye care in rural communities in Ghana, policies focused on the uniform distribution of optometrists need to be enacted. There are existing government policies such as compulsory community service, to address the shortage of healthcare professionals particularly in rural and under-resourced areas. In Ghana, this policy focuses only on nurses whereas in other countries, such as South Africa and Cuba, physicians and other allied health professions are an integral part of such policies [27]. Amendment of this policy in Ghana to include compulsory community service for new graduate optometrists would be a vital step to bridging the gap in access to eye care services in Ghana. Though compulsory community service can improve appreciation of rural services and availability of health workers in the rural areas, it may be flawed by a poorer care by inexperienced caregivers in the underserved areas [28]. More than half of the respondents in our study were opposed to compulsory community service, hence such a policy when being implemented should be cognizant of the possibility of premature vacation from post and discontinuity of patient care [28]. The high objection to compulsory community service may be due to the fact that most of the rural communities lack extrinsic motivational factors including financial incentives, scholarship for further studies, better living conditions and career ladder jump which were identified as the most attractive incentives that would influence graduates to practice in rural settings. It is important to note that implementing measures revolving around these factors would draw more optometrists into rural practice and improve the access and standard of eye care available to these deprived communities.

The strength of this study is that it provides information that could improve the availability and accessibility of eye care services in the rural and remote areas of Ghana. This will help reduce visual impairment in these parts of the country. However, a limitation of the study is that it did not assess the method of financing their education, which could have influenced students’ responses. Another limitation is the quantitative nature of this study which is subject to the shortcomings of a quantitative study such as limited in-depth understanding and investigation of students’ responses.

Readers should also have in mind that social desirability bias; the tendency of survey respondents to answer questions in a manner that will be viewed favorably by others, particularly on the part of the students who hailed from rural areas could have influenced the responses. We, however, believe that the responses provided in this study reflect the reality as per our previous study [7]. Since the views expressed in this paper are only intentions which may well change in the future, there is a need to validate the responses by following up into actual practice between 2 and 3 years after the students have gone through the mandatory one-year internship and are practicing.

## Conclusion

This research has shown that few Ghanaian Optometry students from the urban areas have a desire to practice in rural areas after graduation, with those from rural backgrounds more inclined to return and practice in rural areas. More importantly, optometry students, irrespective of their background may opt to work in rural areas for NGOs or the Government (public sector) after their training. Extrinsic motivational factors would attract graduating optometry students to rural areas, regardless of their origin. The findings of this study highlight the need for Universities offering optometry and Governments/Government Agencies to amend their admission policies and compulsory community services policy respectively, with the view of admitting more students from rural areas and also ensuring equitable distribution of optometrists with emphasis on rural areas respectively. There is also a high need for governments and NGOs (involved in eye care) to ensure that the necessary incentives and policy measures on monitoring and evaluation are implemented to ensure that those who are posted to rural areas do not prematurely vacate post, but commit to improving access to quality eye care with the view of eliminating avoidable blindness, the main goal of Vision 2020.

## Abbreviations

KNUST: Kwame Nkrumah University of Science and Technology
NGO: Non-Governmental Organization
UCC: University of Cape Coast
